# Comparing the Usefulness of the 1997 and 2009 WHO Dengue Case Classification: A Systematic Literature Review

**DOI:** 10.4269/ajtmh.13-0676

**Published:** 2014-09-03

**Authors:** Olaf Horstick, Thomas Jaenisch, Eric Martinez, Axel Kroeger, Lucy Lum Chai See, Jeremy Farrar, Silvia Runge Ranzinger

**Affiliations:** Institute of Public Health, University of Heidelberg, Heidelberg, Germany; Department of Infectious Diseases, Section Clinical Tropical Medicine, Heidelberg University Hospital, Heidelberg, Germany; Instituto de Medicina Tropical, Pedro Kuori, La Habana, Cuba; Liverpool School of Tropical Medicine, Liverpool, United Kingdom; Special Programme for Research and Training in Tropical Diseases, World Health Organization, Geneva, Switzerland; Department of Paediatrics, Faculty of Medicine, University of Malaya, Kuala Lumpur, Malaysia; Wellcome Trust, Oxford, United Kingdom; Consultant in Public Health, Ludwigsburg, Germany

## Abstract

The 1997 and 2009 WHO dengue case classifications were compared in a systematic review with 12 eligible studies (4 prospective). Ten expert opinion articles were used for discussion. For the 2009 WHO classification studies show: when determining severe dengue sensitivity ranges between 59–98% (88%/98%: prospective studies), specificity between 41–99% (99%: prospective study) - comparing the 1997 WHO classification: sensitivity 24.8–89.9% (24.8%/74%: prospective studies), specificity: 25%/100% (100%: prospective study). The application of the 2009 WHO classification is easy, however for (non-severe) dengue there may be a risk of monitoring increased case numbers. Warning signs validation studies are needed. For epidemiological/pathogenesis research use of the 2009 WHO classification, opinion papers show that ease of application, increased sensitivity (severe dengue) and international comparability are advantageous; 3 severe dengue criteria (severe plasma leakage, severe bleeding, severe organ manifestation) are useful research endpoints. The 2009 WHO classification has clear advantages for clinical use, use in epidemiology is promising and research use may at least not be a disadvantage.

## Introduction

The World Health Organization (WHO), with its Special Program for Research and Training in Tropical Diseases (WHO/TDR), issued new dengue guidelines in 2009,^1^ including the 2009 WHO dengue case classification: dengue and severe dengue (D/SD). Warning signs (WS) have been established for triage to help clinicians with symptomatic cases in need of closer surveillance and/or hospitalization (dengue with warning signs [D+WS]).

Historically, the 1997 WHO dengue case classification (dengue fever (DF), dengue hemorrhagic fever (DHF) and dengue shock syndrome (DSS) was developed in 1975 by expert consensus based on studies on Thai children in the 1950s and ≤60s, with modifications in 1986 and 1997.^2^ In the last modification in 1997, four grades of DHF were defined (DHF1, −2, −3, and −4), with DHF1 and −2 being DHF and DHF3 and −4 being DSS.^3^

In this work, we will refer to the dengue case classification recommended by the WHO in 2009 as the D/SD classification and the WHO 1997 classification as the DF/DHF/DSS classification.

The reasons for developing D/SD were the shortcomings of DF/DHF/DSS, which were established in many studies and furthermore, summarized in a systematic review.^2^

DF/DHF/DSS (1) is poorly related to disease severity, (2) misdirects clinicians identifying severe disease, (3) is difficult to use (tests required are often not available/difficult to apply), (4) does not help for triage in outbreaks, and (5) leads to different reporting globally as a result of the difficulties in using the classification for reporting clinicians.

The main emphasis of D/SD is, therefore, to help clinicians to identify and manage cases of severe dengue timely. It helps to save resources and contributes to a reduction of dengue mortality.

Based on the largest prospective multicenter study, the Dengue and Control (DENCO) study,^4^ D/SD describes dengue as it currently occurs globally, focusing on severe dengue, defined as plasma leakage (shock or fluid accumulation with respiratory distress, which includes the former DSS), severe bleeding, or severe organ manifestation. With the improved description of dengue cases, case reporting is facilitated. WS have been empirically validated to some extent in the DENCO study. A larger study is currently under way to evaluate and define the predictive value of WS in outpatients (for the need of hospitalization) and inpatients (for severe disease).^5^ Furthermore, D/SD is based on best available evidence (evidence grade 1/2)^6^ on each step of the development from basic to implementation research.^7^

A discussion evolved internationally on the usefulness and applicability of D/SD compared with DF/DHF/DSS, and in a relatively short period of time, numerous studies have been published by many independent research groups. The objective of this study is to provide a systematic literature review of the studies published, comparing D/SD and DF/DHF/DSS to facilitate the discussion about the usefulness of the different classification systems.

## Methods

Reporting items for systematic reviews and meta-analyses (PRISMA) Statement for systematic reviews and meta-analyses^8^ were followed. This study defines (1) case definition as the description of clinical and laboratory parameters to define a dengue case compared with other febrile illnesses and (2) case classification as the different stages of the spectrum of dengue severity, either D, including D+WS, or SD.

Eligibility criteria included (1) research on dengue case classification and/or dengue case definition, (2) comparison of D/SD and DF/DHF/DSS, (3) any comparative/analytical study design, and (4) published after the publication of the new WHO dengue guidelines.^1^

All languages were included; the search was conducted in English only. Studies looking only at one classification were excluded for failing to compare the different models. Also excluded were studies not performed in dengue-endemic countries. Studies without a defined methodology, including expert opinion and conference proceedings, were collected and used for the discussion.

The literature search and analysis were developed and carried out through July 15, 2013 with two data extractors. The search terms derived from two major categories were (1) dengue disease (dengue) combined with (2) classification and/or definition.

The search strategy was applied to the following databases: US National Library of Medicine and the National Institutes of Health Medical Database (PubMed), the Latin American and Caribbean Health Sciences Database (Lilacs), Excerpta Medica Database (EMBASE), and the Cochrane Database of Systematic Reviews (CDSR). The WHO library database (WHOLIS) and Google Scholar were searched for grey literature. Relevant literature was screened for additional articles in the reference sections.

Because of the limited search options available to this field of research, a very broad search strategy was used, using a reduced number of combinations to increase the number of initial hits.

All results were screened for duplicates. In the next stage, results were screened based on the title and abstract only, and the full texts of potentially relevant studies were subsequently assessed. Relevant information was tabulated in evidence tables.

Study designs were divided into the following categories: (1) prospective studies of dengue cases, (2) studies analyzing *post-hoc* existing prospectively collected databases of dengue cases, (3) studies reviewing retrospectively existing medical charts of dengue cases, and (4) acceptability studies (mostly of qualitative design).

No studies were excluded in the analysis for quality reasons if the eligibility criteria were met, but limitations and possible biases are reported in the results section. The analysis followed the categories according to the use of the case classification: (1) clinical use (establishing severe dengue disease using WS for identifying severe dengue disease, detecting dengue, usefulness for triage, usefulness for outbreaks, and ease of application by clinicians), (2) use in surveillance detecting dengue cases and detecting dengue outbreaks as well as reporting of the different levels of severity of dengue disease (D/SD), and (3) use in research.

## Results

In total, 782 studies were identified during the electronic search that were potentially relevant to the research question, including duplicates. After the screening of titles and abstracts, 25 studies remained eligible.^10^–^34^ Eligible studies are tabulated in Table 1. Ten studies^22^–^31^ were relevant to the research question based purely on expert opinion, without stating a particular methodology as to how the results were derived. These studies were excluded from the analysis but reflected in the discussion for the purpose of highlighting the different opinions. Three additional studies were excluded after full assessment of the text, because they only assessed DF/DHF/DSS without comparison with D/SD^32^,^33^ or dealt with a particular subgroup of cases not representative of dengue-endemic countries (dengue in travellers^34^).

**Figure 1.**
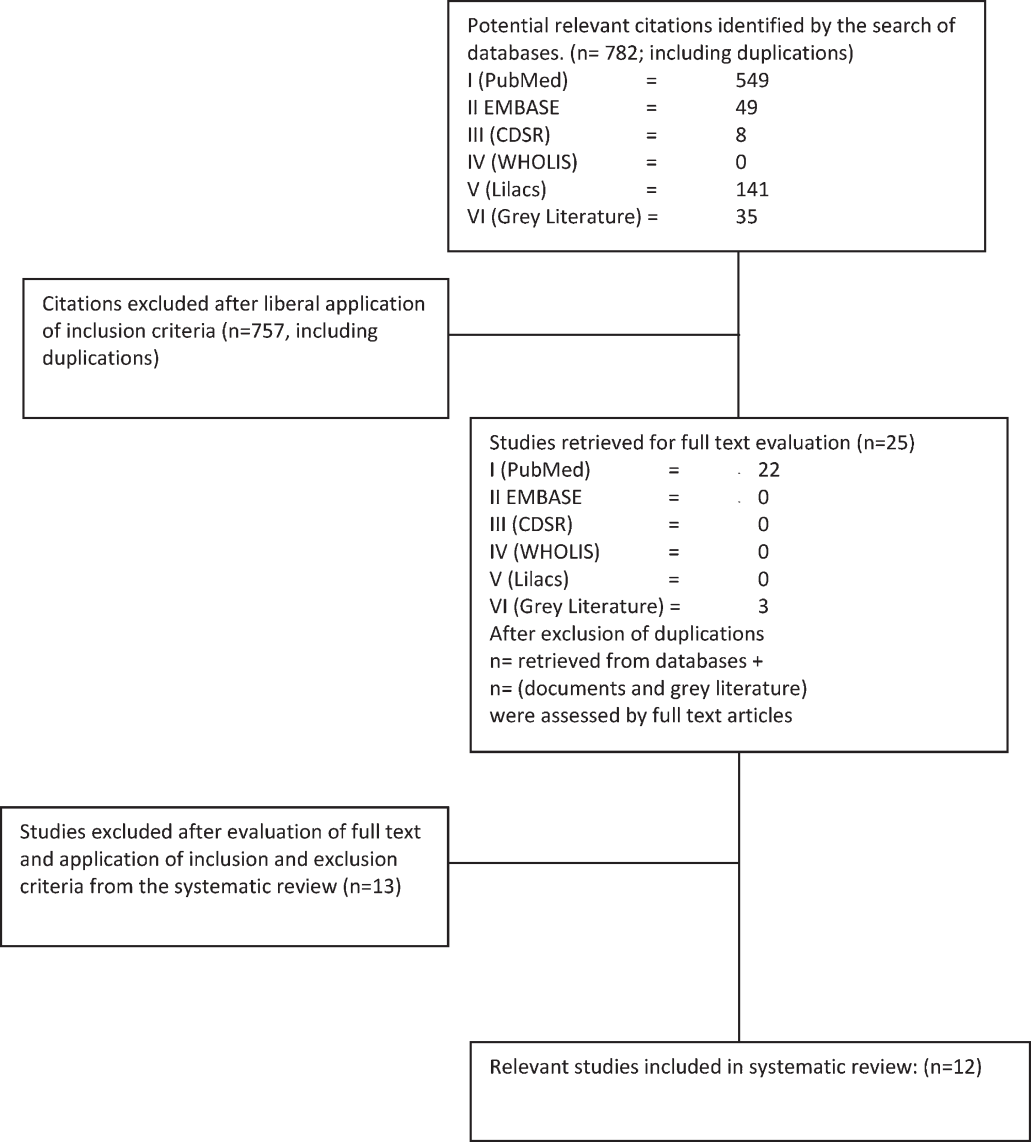
Flowchart of the systematic search for literature.

Of the remaining 12 studies,^10^–^21^ 11 studies were from published databases, and one study was from grey literature. No other studies were identified from the reference lists. Figure 1 summarizes the process of selection in a flowchart.

All studies were published after 2009—the date set for initiating the search. Most of the studies included (prospective, *post-hoc* analysis of existing datasets or review of existing medical charts, and any qualitative design) were performed in Asia, with the exception of three studies: one study that included 18 study sites worldwide,^10^ one study from Nicaragua,^16^ and one study from Peru.^18^ Studies^10^–^13^ are prospective studies published between 2010 and 2013. All other studies used datasets either prospectively developed and compared *post-hoc* with the use of D/SD and DF/DHF/DSS or retrospectively derived from case notes of dengue-positive cases developed over time and without a strict study protocol. One study^10^ also included the acceptability of the different dengue case classifications with qualitative methods.

Sample sizes of the studies^10^–^21^ were relatively small, ranging from 50 to 300 cases (mostly less than 100 cases), with the exception of three studies^10^,^17^,^20^ with more than 1,000 cases each. Most of the studies included adults and children; only three studies were confined to pediatric centers.^12^,^16^,^21^

All studies were performed in hospital settings of either secondary or tertiary level hospitals. The study in ref. 10—with 18 study sites—had the biggest scale in case numbers and the most comprehensive scope with all levels of healthcare delivery systems included in Asian as well as Latin American countries.

Studies often addressed multiple questions; the following results are presented according to the categories described in the methods sections. In general, the prospectively collected studies are largely in favor of the use of D/SD in the clinical setting; the retrospective studies have similar results, apart from the study in ref. 15

### Clinical use.

#### Which classification describes the clinical picture of dengue cases better?

The studies dealing with this question all agree that D/SD classifies dengue as it occurs clinically. The study in ref. 10 in 18 countries with the biggest sample overall concludes that “13.7% of cases could not be classified according to DF/DHF/DSS (missing data e.g. haematocrit, platelet counts, and tourniquet tests on > 50% of charts), compared to 1.6% for D/SD. 32.1% of severe dengue cases could not be classified in the DF/DHF/DSS system.” The study in ref. 17, also in a large sample, reconfirms that “DF/DHF/DSS and D/SD were discordant in defining severe disease (p: 0.001).” D/SD describes severe disease, whereas DF/DHF/DSS describes severe disease less well. Similarly, findings in the study in ref. 19 are “a cross tabulation [that] showed DF cases were distributed in all of the severity groups stratified by D/D+WS/SD (53.8% Group D/45.4% Group D+WS/0.8% Group SD). Of the DHF cases, 23 (79%) were categorized as Group D+WS, and six (20.7%) as Group SD.”

When looking at quality of studies, the studies in refs. 10, 17, and 19 of this systematic literature review provide probably the best available quantitative evidence for this question.

#### Identifying dengue (case definition).

Only one study dealt with the question of whether the case definitions for D are more useful to detect dengue compared with DF.^14^ The results—retrospectively based on 164 dengue cases compared with 200 other febrile illnesses (OFIs)—show for DF (as defined by the DF/DHF/DSS classification) a sensitivity of 95.4% and a specificity of 36.0% and for D (as defined by the D/SD classification) a sensitivity of 79.9% and specificity of 57.0%. The study acknowledges that D/SD was not primarily designed for detecting dengue. When using an NS1 test, the sensitivity and specificity for both dengue case classifications improve considerably (Table 1).

#### Identifying severe disease (case classification).

Several studies looked at sensitivity and specificity of D/SD and DF/DHF/DSS for identifying severe dengue. Sensitivity for detection of severe disease has been calculated by five studies,^11^,^13^,^16^,^18^,^21^ with results in the range from 59% to 98% for SD (as defined by the D/SD classification). The prospective studies^11^,^13^ estimate sensitivity at 88% and 98%, respectively. Specificity is estimated between 41% and 99% (99% for the prospective study presenting values^11^). Sensitivity and specificity for DHF (as defined by the DF/DHF/DSS classification) range between 24.8% and 89.9% and between 25% and 100%, respectively (sensitivity of 24.8% and 74% for the prospective studies and 100% specificity for the prospective study presenting data) (Table 2).

In summary, comparing DF/DHF/DSS with D/SD, it seems that, in most studies, D/SD turned out to be better in terms of sensitivity and specificity for distinguishing D and SD, which is summarized in the study in ref. 10 (here derived from qualitative data: “the revised classification also proved to be more sensitive for timely recognition of severe disease”).

#### WS for SD disease.

Several studies looked at the value of WS as predictors of severe disease. The study in ref. 11 suggests that having at least five WS may be a good predictor of severe disease; in particular, lymphocyte counts < 1,500 cells/mm^3^, platelet counts < 20,000/mm^3^, and raised AST levels were associated with SD. However, in the study in ref. 20, the three most common WS were lethargy, abdominal pain/tenderness, and mucosal bleeding. Also, no single WS alone or combined had a sensitivity of more than 64% in predicting severe disease.^20^ Specificity was 90% for both DHF and SD with persistent vomiting, hepatomegaly, hematocrit rise, and rapid platelet drop.^20^

In practice, however, it seems that WS are significant events that are well-documented in medical charts,^10^ but using WS as compulsory hospitalization criteria may well increase workload.

#### Usefulness for triage and in outbreaks.

The study in ref. 10 finds—with focus groups and questionnaires among clinicians in 18 countries—that D/SD “was easily applicable in clinical practice, also seen to be useful for triage and case management by medical staff, more frequently than the DF/DHF/DSS classification.” The study in ref. 15 analyses medical charts of 274 confirmed dengue cases. When using DF/DHF/DSS, more intensive treatment (with careful monitoring and intravenous rehydration) was needed in 94 cases compared with 189 cases when using D+WS and SD as criteria. Similarly, the study in ref. 17 calculates the requirement for hospitalization increasing from 17.0% to 51.3% using D/SD. This finding is in agreement with the findings in the study in ref. 18: “DF/DHF/DSS may save health care resources with its more stringent definition of a DHF case requiring the most intensive hospital intervention.” However, the same study points out that “the use of D/SD guidelines in this outbreak facilitated the early admission of those with life threatening dengue disease for appropriate clinical management.”

#### Ease of application by clinicians.

Only two studies assessed the ease of application by health personnel, with positive results overall for the D/SD classification: the study in ref. 10 shows, with questionnaires and focus groups, that D/SD was easily applicable in clinical practice more frequently than the DF/DHF/DSS classification; the former was also seen to be useful for triage and case management by medical staff. The study in ref. 16 analyzed with interrater agreement methods how clinicians agreed with each other when classifying the same cases according to either D/SD or DF/DHF/DSS; the former had substantial agreement (κ = 0.62, *P* = 0.001), and the latter had moderate agreement (κ = 0.46, *P* = 0.001).

### Use in surveillance and research.

No study examined the use of D/SD compared with DF/DHF/DSS in the context of dengue surveillance and/or reporting; the study in ref. 10 argued that, with the improved description of cases and especially, the improved sensitivity for severe cases, data collection for epidemiological returns should be improved. However, the study in ref. 16 concludes that the use in epidemiology needs to be evaluated.

No study addressed whether D/SD has advantages or disadvantages for research. The study in ref. 16 states that its use in pathophysiological and epidemiological studies needs additional evaluation.

Because there were no studies identified that formally evaluated D/SD compared with DF/DHF/DSS in these areas, we refer to the discussion section below, where expert opinions/viewpoints were also included.

### Additional results.

Most studies conclude that additional research needs to address unsolved questions, especially in larger studies (locally adapted case definitions for diagnosing dengue in the absence of confirmatory laboratory testing and the predictive value of WS in outpatients and inpatients) and different settings.^11^,^12^

It has also been emphasized that training needs to be addressed as well as the development of local clinical protocols for dengue.

## Discussion

### Limitations.

Because D/SD was only introduced in 2009, the number of studies available is surprising, probably underlining the importance of this question. Nevertheless, the number of studies available for answering the study question was limited, and therefore, all available data in this study, including lower-quality studies and retrospective studies, had to be considered in this review. Only very few prospective studies are available, and even fewer qualitative studies have been reported. Regarding the quality of retrospective studies, it is often unclear if all data are available retrospectively to conduct such studies. Also, almost all studies reviewed are on a secondary or tertiary level of care and not primary care. Most studies are not in resource-poor settings. Furthermore, sample sizes are mostly small, and data from Asian countries prevail.

Publication bias is another concern, especially for the expert opinion papers; therefore, they have been excluded from the analysis and are mentioned only in the discussion.

### Relative value of the two dengue case classifications.

The first question is which purpose does the classification serve: clinical care, surveillance, or research (see also ref. 29). In this section, we will consider these three main areas of application of the dengue case classification.

For the clinical use of D/SD, the three prospective studies dealing with this question confirm that D/SD has advantages in defining the severity of dengue cases.^10^,^11^,^13^ The evidence is even stronger when considering sample size. The study in ref. 10 is, by far, the biggest study in this context, conducted globally in 18 different dengue-affected countries, and the study in ref. 11 is a medium-sized study. The sensitivity and specificity analyses^11^,^13^,^16^,^18^,^21^ are equally in favor of D/SD. However, this finding is no surprise, because the classification has been designed^4^ “to develop a revised evidence-based classification that would better reflect clinical severity.”

The ability to distinguish D and SD—as it currently occurs globally—is probably the biggest advantage of D/SD.

Expert opinion underlines this finding. The studies in refs. 26 and 29 argue—mainly with reference to the study by Alexander and others^4^ and the study in ref. 10—that D/SD offers a better categorization than the DF/DHF/DSS classification.

In a letter responding to study,^13^ it has been queried whether the classification is useful for predicting dengue severity and also, if the application of the classification can be considered to have an easy application.^31^

Clinical use of the classification also includes the use of WS. It would be beneficial to have robust and validated WS, but the level of evidence for most WS as established in D/SD is not very high, because large quantitative studies are needed to establish the validity of single parameters and/or combinations of parameters in predicting the progress to SD. This finding has been acknowledged by the two quantitative studies dealing with this question^12^,^20^ and the expert opinions: responding to the study in ref. 20, it has been mentioned that convincing studies about WS need to be prospective and should include primary care.^30^ The WS, as defined in the D/SD framework, were considered to require more specific definitions.^22^ Currently, large global prospective studies are under way to help improve the knowledge of the value of WS (International Research Consortium on Dengue Risk Assessment, Management and Surveillance study (IDAMS) at http://ichgcp.net/clinical-trials-registry/NCT01550016 and Laboratory Diagnosis and Prognosis of Severe Dengue study at http://ichgcp.net/clinical-trials-registry/NCT01421732).

The dengue case definition is important for diagnosing a dengue case, especially when diagnosing without laboratory confirmation in resource-poor settings.^14^ The D/SD framework uses the same criteria for case definitions as defined by the DF/DHF/DSS framework (which is based on expert opinion), with some modifications also driven by expert opinion. However, it is well-known that using only clinical criteria for discriminating between different febrile illnesses is difficult or impossible. Similar to the WS, this question can only be addressed with large prospective global studies, which are under way (IDAMS study above).

This finding leads to the question of whether a clinically based dengue diagnosis and the application of WS can be useful for triage. Some studies, based on qualitative arguments^10^ and expressing the viewpoints of clinicians, come to a positive answer; others refer to the fact that the ease of application is also documented in studies.^16^ One retrospective study argues that the caseload for hospitalization increases dramatically.^15^ However, the same study acknowledges that only 68% of cases with SD disease can be classified with the DF/DHF/DSS framework. In other words, 32% of cases are not classifiable and maybe missed, reflecting the low sensitivity of DHF. This result has been summarized in the study in ref. 18, which acknowledges that the workload may potentially increase with the broad use of WS but that cases with severe disease are correctly identified. It seems that the application of the WS in the algorithm needs adjustment to the locally available resources, which was mentioned in the WHO guidelines^1^: “depending on the clinical manifestations and other circumstances, patients may … be referred for in-hospital management (Group B).” Whether increased admissions with early treatment may reduce severe/fatal illnesses needs to be analyzed as well.

The expert opinion papers vary on this issue. One study^22^ supports the view that D/SD is beneficial for triage; another study^31^ reports the excellent experience of using D/SD for grouping of patients for additional management.

It would be useful to see more studies using the algorithms for treatment as suggested in the WHO guidelines^1^ and the *Clinical Handbook on Dengue Management*,^9^ with local adaptations in prospective studies—maybe in outbreak situations—and use of qualitative methods to assess the usefulness of D/SD in real-life circumstances.

No quantitative studies assessed the use of the D/SD versus the DF/DHF/DSS classification in dengue surveillance and case reporting. However, the difficulties in applying DF/DHF/DSS led to the development of multiple local adaptations and put global comparability at risk. Studies assessing the use of D/SD in clinical settings argued that the improved description of dengue as it occurs globally, leading to more specific categorizations according disease severity, should lead to improved reporting. Likewise, the opinion-based papers in this systematic review argue that D/SD^23^ “maybe useful for surveillance and reporting” and provide the opportunity of improved international comparison of data.^26^ However, to confirm this answer, additional research is warranted.

For the use of the D/SD classification as an outcome in basic research or pathogenesis research, no quantitative data were available.

A viewpoint/opinion paper^24^ argues that “pathogenesis research should be conducted as much as possible on carefully defined categories of human disease response. This requires splitting, not lumping.” This work was responded to by a group of authors^29^ arguing that “DF/DHF/DSS was too complicated to use in clinical or public health settings, yet was not sufficiently precise for detailed pathogenesis studies” and that “D/SD brings clarity, clinical and epidemiological utility, and the potential for development of more precise definitions of clinical phenotype for pathogenesis studies.” Therefore, D/SD, with its three severity markers—severe plasma leakage, severe bleeding, and severe organ manifestation—can be further refined for basic research.

Two other views were expressed: (1) quantitative studies confirmed that the DF/DHF/DSS framework does not reflect levels of severity^10^,^17^,^19^; and (2) critical evaluation of D/SD is limited to the use in basic research; however, this critical evaluation is not based on specific studies, but is based on expert opinion only.^23^,^24^,^27^

Some studies suggest solutions—based on their analysis—to reclassify the dengue disease classifications (for example, introducing subgroups for dengue with organ failure). However, it has to be underlined that these suggested solutions are based on each individual study presenting individual solutions^15^,^17^ and not the result of the highest available evidence for the particular study question, the latter being one of the strengths of D/SD.^25^

In conclusion, this systematic review confirms—almost 5 years after its introduction—that the D/SD classification is able to detect disease severity with high sensitivity, thus assisting clinical management and potentially, contributing to reducing dengue mortality.

The limitation of not having more appropriate studies available is also a result of this study, with the urgent call for studies to be designed prospectively and include primary care.

Diagnosis of dengue by clinical parameters only continues to be a challenge. It is recommended to await the findings of the ongoing large clinical studies for a possible adaptation of case definitions and WS. Furthermore, it is recommended to study the performance of D/SD for triage, especially in outbreak situations. For surveillance and global reporting, a unified classification system with accurate information on disease severity would be advantageous.

For pathogenesis research, the D/SD classification may open new opportunities, with a fresh look at underlying pathology now that the spectrum of disease is better described.

## Figures and Tables

**Table 1:** Included and excluded studies

Ref.	Authors	Year	Study type	Focus	Large/small*	Prospective/retrospective	Country	Main research question	Methods	Results	Conclusion
Prospective studies (follow-up of dengue cases)											
10	Barniol and others	2011	Mixed methods, three arms: (1) quantitative (prospective and retrospective chart reviews), (2) qualitative (health personnel questionnaire), and (3) focus groups	Usefulness and applicability of the new dengue case classification	Large	Prospective, some retrospective data	Global: 18 countries	Comparison of D/SD and DF/DH/DSS: (1) clinical applicability; (2) triage and management usefulness; (3) user-friendliness and acceptance	(1) Prospective/retrospective medical chart reviews; (2) Self-applied questionnaires; (3) focus groups	13.7% of cases could not be classified according to DF/DHF/DSS (missing data; e.g., hematocrit, platelet counts, and tourniquet tests on > 50% of charts) compared with 1.6% for D/SD. WS for SD (necessary for the D+WS category) were documented in a large proportion. The revised classification also proved to be more sensitive for timely recognition of SD. 32.1% of SD cases could not be classified in the DF/DHF/DSS system. D/SD was easily applicable in clinical practice, also seen to be useful for triage and case management by medical staff more frequently than the DF/DHF/DSS classification. Concerns in questionnaires and focus groups included (1) hospitalization rates might increase if the WS are not precisely defined and warrants additional studies, (2) cost implications if more patients are being admitted, and (3) training needs, dissemination, and concise clinical protocols.	The revised dengue classification has a high potential for facilitating dengue case management and surveillance, being more sensitive, especially for severe disease, more accepted, and user friendly.
11	Basuki and others	2010	Prospective study of dengue cases	SD	Medium	Prospective	Indonesia	Comparison of D/SD and DF/DH/DSS	Quantitative analysis of prospectively followed up dengue cases, with clinical and laboratory parameters; sensitivity and specificity testing	Of 145 cases, using a non-severity definition, 122 cases (84.1%) were classified as non-severe, of which using DF/DHF/DSS, 70 (48.3%) were classified as DF, 39 (26.9%) were classified as DHF grade I, 13 (95) were classified as DHF grade II. 23 (15.95) were classified as severe, of which 16 (11%) were classified as DHF grade III and 7 (4.8%) were classified as DHF grade IV. With clinical interventions included, eight cases (6.6%) originally classified as having non-SD infection were reclassified as having severe infection (sensitivity = 74%, specificity = 100%, likelihood ratio (−) = 0.26). Using D/SD, 117 cases (80.7%) were classified as non-severe, of which 79 (54.5%) were classified as D–WS, 38 (26.2%) were classified as D+WS, and 28 (19.3%) were classified as SD. Using clinical intervention, four cases (3.4%) that were originally non-severe were reclassified as SD (sensitivity = 88%, specificity = 99%, likelihood ratio (+) = 98.88, likelihood ratio (−) = 0.13). Binary logistic regression: SD was better detecting SD (*P* = 0.000, Wald = 22.446) than DF/DHF/DSS (*P* = 0.175, Wald = 6.339).	SD may be better at detecting SD infection cases compared with DF/DHF/DSS. Additional research is needed with larger numbers of cases in multiple centers using the revised clinical management guidelines.
12	Jayaratne and others	2012	Prospective study of dengue cases	WS	Medium	Prospective	Sri Lanka	(1) Usefulness of WS predicting SD; (2) define other simple laboratory parameters useful in predicting SD	Quantitative analysis of prospectively collected dengue cases with clinical and laboratory parameters.	In 184 cases in two tertiary centers, five or more WS are significantly (*P* = 0.02) associated with the development of SD (odds ratio = 5.14, 95% confidence interval = 1.312–20.16). AST levels were significantly higher (*P* = 0.0001) combined with abdominal pain (mean = 243.5, SD ± 200.7) compared with non-abdominal pain (mean = 148.5, SD ± 218.6). High AST levels were also significantly associated (*P* < 0.0001) with SD (odds ratio = 27.26, 95% confidence interval =1.632–455.2). Lymphocyte counts < 1,500 cells/mm^3^ were significantly (*P* = 0.005) associated with SD (odds ratio = 3.367, 95% confidence interval = 1.396–8.123). Platelet counts < 20,000cells/mm^3^ were again significantly associated (*P* < 0.001) with SD (odds ratio = 1.632–455.2, 95% confidence interval = 3.089–14.71).	The presence of five or more WS seems to be a predictor of SD. Lymphocyte counts < 1,500 cells/mm^3^, platelet counts < 20,000/mm^3^, and raised AST levels were associated with SD and could be used to help identify patients who are likely to develop SD.
13	Prasad and others	2013	Prospective study of dengue cases	SD	Small	Prospective	India	To assess the accuracy and applicability of the revised WHO classification (2009) of dengue in children seen at a tertiary healthcare facility in India	Quantitative analysis of prospectively collected dengue cases with clinical and laboratory parameters; sensitivity and specificity testing	56 patients tested positive for dengue; 5 patients (8.9%) received level 1 treatment, 10 patients (17.8%) received level 2, and 41 patients (73.2%) received level 3. 42 (75%) patients were classified as DF, and 13 patients (23.2%) were classified as DHF/DSS; one patient was unclassifiable. 1 patient (1.7%) was classified as D, 9 patients (16%) were classified as D+WS, and 46 patients (82.1%) were classified as SD. Many of the severe manifestations (encephalopathy, shock, mucosal bleed, platelet count < 20,000, respiratory distress, liver enzymes > 1,000 U/L) were seen in cases classified as DF, whereas these cases were mostly classified as SD. Sensitivity was 24.8% for DF/DHF/DSS and 98% for SD.	SD has very high sensitivity for identifying SD and is easy to apply.
Analysis in existing dataset (prospectively collected dengue cases with *post-hoc* analysis or retrospective analysis of existing medical charts)											
14	Chaterji and others	2011	*Post-hoc* analysis using an existing prospectively collected dataset	Early dengue detection	Medium	Analysis using an existing prospectively collected dataset	Singapore	(1) Sensitivity/specificity of NS1 strip with DF/DHF/DSS and D/SD for diagnosis of acute dengue; (2) sensitivity of the tests in primary compared with secondary dengue, virus serotype, and clinical characteristics observed in the early stages of dengue illness	Retrospective quantitative analysis of laboratory and clinical parameters in existing dataset; sensitivity and specificity testing	Of 354 cases diagnosed definitely as 154 dengue and 200 OFI cases, DF/DHF/DSS criteria correctly diagnosed 147 (95.4%) dengue cases and labeled 128 (64%) as dengue (sensitivity of 95.4% and specificity of 36.0%). D/SD detected 123 (79.9%) of dengue cases and 86 (43.0%) of OFI cases (sensitivity of 79.9% and specificity of 57.0%). The NS1 strip had a sensitivity and specificity of 77.3% and 100%, respectively, at 15 minutes and 80.5% and 100%, respectively, at 30 minutes.	In conclusion, the 1997 WHO dengue case definition can be useful in ruling out dengue, whereas the dengue NS1 Ag strip can be used as a bedside diagnostic test to support a diagnosis of acute dengue, although caution is advised in the interpretation of this test in dengue hyperendemic areas.
15	Kalayanarooj and others	2011	Retrospective application of D/SD on existing medical charts	Case management	Medium	Retrospective analysis using existing medical charts	Thailand	(1) Comparing DF/DHF/DSS and D/SD for clinical management; (2) assessing four criteria of the DHF case definition for possible modification	Retrospective quantitative analysis of laboratory and clinical parameters in existing medical charts; sensitivity and specificity testing	274 confirmed dengue patients and 24 non-dengue febrile illnesses (ND): 180 DF (65.7%), 53 DHF grade I (19.3%), 19 DHF grade II (6.9%), 19 DHF grade II (6.9%), and 3 DHF grade IV (1.1%); 85 D (31%), 160 DW (58.4%), and 29 SD (1.1%). Therewere eight DSS patients who had AST > 1,000 U and one patient who presented with encephalopathy not classifiable by DF/DHF/DSS. One non-dengue patient who presented with gastrointestinal bleeding was classified as SD. At least one of the WS was found in 50% of ND cases, 53.3% of DF, 83% of DHF grade I, 88.2% of DHF grade II, 100% of DHF grade III, and 100% of DHF grade IV. Vomiting and abdominal pain were the two most common WSS found in both ND and dengue patients. Bleeding and/or positive tourniquet test were found in 69.7% of DHF patients. Hemoconcentration could detect plasma leakage in 44.7%, and CXR added up evidence of plasma leakage to 86.3%. Ultrasonography was the most sensitive technique to add evidence of plasma leakage up to 100%. Platelets ≤ 100,000 cells/mm^3^ were found in 93.5% of DHF patients. Intensive monitoring and careful medical and intravenous fluid management were needed for 94 DHF patients compared with 189 D+WS and SD patients.	DF/DHF/DSS is recommended for use, compared with D/SD, because the latter creates double the workload and needs confirmatory tests. DF/DHF/DSS needs modification: plasma leakage as the major criteria, tourniquet test positive or bleeding symptoms as minor criteria, and add unusual dengue as category.
16	Narvaez and others	2011	*Post-hoc* analysis using an existing prospectively collected dataset; interrater agreement	SD; applicability	Small	Retrospective analysis of an existing prospectively collecteddataset	Nicaragua	Evaluating DF/DHF/DSS and D/SD against clinical intervention levels for diagnosing severity (data from 5 years [2005–2010] of a hospital-based study of pediatric dengue)	Retrospective quantitative analysis of laboratory and clinical parameters in existing dataset; sensitivity and specificity analysis and interrater agreement when applied by different clinicians	Sensitivity and specificity of DF/DHF/DSS for the detection of severe cases of dengue was 39.0% and 75.5%, respectively; sensitivity and specificity of D/SD was 92.1% and 78.5%, respectively. Evaluation of physicians' clinical diagnosis resulted in moderate agreement (κ = 0.46, *P* = 0.001) with the traditional classification and substantial agreement (κ = 0.62, *P* = 0.001) with the revised classification.	D/SD is most useful for the physician for the detection of severe cases of dengue, but its use in pathophysiological and epidemiological studies needs additional evaluation in future research.
17	Gan and others	2013	*Post-hoc* analysis using an existing prospective dataset of confirmed dengue cases	Describing SD and use/application in the clinical setting	Large	Retrospective analysis of an existing prospectively collected dataset	Singapore	Evaluating D/SD and DF/DHF/DSS using laboratory-confirmed adult dengue cases in two epidemics in Singapore: 2004 (predominantly dengue serotype 1) and 2007 (predominantly dengue serotype 2)	Retrospective quantitative analysis of laboratory and clinical parameters in existing dataset; sensitivity and specificity analysis	1,278 dengue confirmed cases. DHF occurred in 14.3%, DSS occurred in 2.7%, and SD occurred in 16.0%. DF/DHF/DSS and D/SD were discordant in defining severe disease (*P* = 0.001). Five DSS patients (15%) were classified as non-SD without WS. Of SD patients, 107 did not fulfill DHF criteria (14.9% had self-resolving isolated elevated aminotransferases, 18.7% had gastrointestinal bleeding without hemodynamic compromise, and 56.1% had plasma leakage with isolated tachycardia). Comparing against requirement for intensive care, including the single death in this series, all six had SD; only four had DHF, because two lacked bleeding manifestations but had plasma leakage. Increasing length of hospitalization was noted among severe cases with both classifications, but the trend was only statistically significant for D/SD. Length of hospitalization was significantly longer for severe plasma leakage compared with severe bleeding or organ impairment. Requirement for hospitalization increased using D/SD from 17.0% to 51.3%.	D/SD is clinically useful. It retains criteria for plasma leakage and hemodynamic compromise from DF/DHF/DSS and refined definitions of severe bleeding and organ impairment. Findings from our retrospective study may be limited by the study site—a tertiary referral center in a hyperendemic country—and should be evaluated in a wider range of geographic settings.
18	Siles and others	2013	Retrospective analysis using existing medical charts	SD	Small	Retrospective	Peru	To evaluate the usefulness of DF/DHF/DSS and D/SD	Retrospective quantitative analysis of laboratory and clinical parameters in existing medical charts; sensitivity and specificity analysis	Sensitivity of DF/DHF/DSS and D/SD in capturing patients with life-threatening disease was 0% and 59%, respectively, and specificity of the two classifications was 98% and 41%, respectively.	DF/DHF/DSS may save healthcare resources with its more stringent definition of a DHF case, which requires the most intensive hospital intervention; use of D/SD guidelines in this outbreak facilitated the early admission of those patients with life-threatening dengue disease for appropriate clinical management.
19	Tsai and others	2012	Retrospective analysis using existing medical charts	SD	Medium	Retrospective	Taiwan	We compared differences in clinical/laboratory features between patients separately classified as DF/DHF and in group D/D+WS/SD	Retrospective quantitative analysis of laboratory and clinical parameters in existing medical charts	148 adult patients (119 DF/29 DHF; 64 D/77 group D+WS/7 group SD) were included. Compared with DF, significantly younger age, lower hospitalization rate, and higher platelet count were found in group D. Compared with DHF, higher platelet count was found in group D+WS. Six of seven patients (86%) classified as group SD fulfilled the criteria of DHF. A cross-tabulation showed that DF cases were distributed in all of the severity groups stratified by D/D+WS/SD (53.8% group D/45.4% group D+WS/0.8% group SD). Of the DHF cases, 23 (79%) were categorized as group D+WS, and 6 (20.7%) were categorized as Group SD. All patients in Group D fell into the category DF.	D/SD effective in identifying SD cases. Heterogeneity in severity suggests that careful severity of discrimination in patients classified in group D+WS is needed. Our data suggest that it is safe to treat patients classified as group D on an outpatient basis.
20	Thein and others	2013	*Post-hoc* analysis in existing prospectively collected dataset	WS	Large	Retrospective	Singapore	Performance of WS for predicting DHF and SD in adult dengue	Retrospective quantitative analysis of laboratory and clinical parameters in existing prospectively collected dataset; sensitivity and specificity analysis in relation to WS	Of 1,507 cases, DHF occurred in 298 (19.5%) and SD occurred in 248 (16.5%) cases. Of these cases, WS occurred before DHF in 124 and before SD in 65 at median of 2days before DHF or SD. Three most common WS were lethargy, abdominal pain/tenderness, and mucosal bleeding. No single WS alone or combined had sensitivity of 64% in predicting severe disease. Specificity was 0.90% for both DHF and SD with persistent vomiting, hepatomegaly, hematocrit rise and rapid platelet drop, clinical fluid accumulation, and any three of four WS. Any one of seven WS had 96% sensitivity but only 18% specificity for SD.	No WS was highly sensitive in predicting subsequent DHF or SD in our confirmed adult dengue cohort. Persistent vomiting, hepatomegaly, hematocrit rise, rapid platelet drop, and clinical fluid accumulation as well as any three or four WS were highly specific for DHF or SD.
21	Van de Weg and others	2012	*Post-hoc* analysis in existing prospectively collected dataset	Disease severity	Small	Retrospective	Indonesia	To evaluate DF/DHF/DSS and D/SD against disease severity	Retrospective quantitative analysis of laboratory and clinical parameters in existing prospectively collected dataset; sensitivity and specificity analysis	D/SD, 69 patients (39.9%) D and 104 patients (60.1%) SD. DF/DHF/DSS: 24 patients (13.9%) DF and 149 patients (86.1%) DHF/DSS. SD: 64 severe plasma leakage, 6 severe bleeding, 18 plasma leakage and bleeding, and 16 severe organ impairment. D: 38 patients (55.1%) had received ITU treatment compared with 13 patients (54.2%) classified as DF. SD 91 patients (87.5%) had received intensive treatment intervention, a slightly higher number than 116 patients (77.9%) in the DHF/DSS group. D/SD specificity, 70.5%; sensitivity, 70.5%. DF/DHS/DSS specificity, 25%; sensitivity, 89.9%	Taken together, we conclude that, in both clinical and research settings, the performance of D/SD is an improvement to DF/DHF/DSS, although more validated and detailed classification criteria need to be defined.
Excluded expert opinion studies relevant to the subject and used in the discussion											
22	Hadinegoro	2012	Review	Comparing DF/DHF/DSS and D/SD for clinical application	N/A	N/A	Indonesia	Not specified	Not specified	Although the revised scheme is more sensitive to the diagnosis of SD and beneficial to triage and case management, there remain issues with its applicability. It is considered by many to be too broad, requiring more specific definition of WS.	Quantitative research into the predictive value of these WS on patient outcomes and the cost-effectiveness of the new classification system is required to ascertain whether the new classification system requires additional modification or whether elements of both classification systems can be combined.
23	Halstead	2012	Review	Comparing DF/DHF/DSS and D/SD for clinical application and pathogenesis	N/A	N/A	N/A	To describe the usefulness of the 1997 and 2009 case classification in relation to underlying pathogenesis	None specified	The WHO 2009 case classification is not useful for clinical work and especially not for pathogenesis research but maybe useful for reporting.	The development of a robust case classification is the priority.
24	Halstead	2013	Review	Comparing the DF/DHF/DSS and D/SD for clinical application and pathogenesis	N/A	N/A	N/A	We discuss the impact that the widespread adoption of the 2009 WHO case definitions may have on the development of research hypotheses or the conduct of research on dengue diseases	None specified	Much of the framework for understanding the etiology of dengue disease, the phenomenon of antibody-dependent enhancement of infection, and concepts of T cell immunopathology has developed out of studies on the dengue vascular permeability syndrome. As illustrated below, if the future pathogenesis research is based on clinical responses included in SD, such patients will exhibit an a mixture of dengue disease syndromes and/or complications of treatment, such as (1) the distinct syndromes contained within the clinical category severe bleeding and (2) inclusion of clinical endpoints that may confuse natural with iatrogenic evolution of disease.	Pathogenesis research should be conducted as much as possible on carefully defined categories of human disease response, which requires splitting, not lumping.
25	Horstick and others	2012	Review	Evidence base	N/A	N/A	N/A	Reviewing the development of D/SD	Review	Step 1: Systematic literature review highlighted the shortcomings of the DF/DHF/DSS: (1) difficulties in applying the criteria for DHF/DSS; (2) the tourniquet test has a low sensitivity for distinguishing between DHF and DF; and (3) most DHF criteria had a large variability in frequency of occurrence. Step 2: An analysis of regional and national dengue guidelines: need to re-evaluate and standardize guidelines; actual ones showed a large variation of definitions, an inconsistent application by medical staff, and a lack of diagnostic facilities necessary for the DHF diagnosis in frontline services. Step 3: A prospective cohort study in seven countries, a clear distinction between SD (defined by plasma leakage and/or severe hemorrhage and/or organ failure) and (non-severe) dengue can be made. Step 4: Three regional expert consensus groups in the Americas and Asia concluded that dengue is one disease entity with different clinical presentations and often, unpredictable clinical evolution. Step 5: Global expert consensus meeting at WHO in Geneva, Switzerland—the evidence collected in steps 1–4 was reviewed, and a revised scheme was developed and accepted, distinguishing D/SD. Step 6: In 18 countries, the usefulness and applicability of D/SD compared with the DF/DHF/DSS scheme were tested, showing clear results in favor of the revised classification (note ref. 1). Step 7: Studies are under way on the predictive value of WS for SD.	The analysis has shown that D/SD is better able to standardize clinical management, raise awareness about unnecessary interventions, match patient categories with specific treatment instructions, and make the key messages of patient management understandable for all healthcare staff dealing with dengue patients. Furthermore, the evidence-based approach to develop prospectively the dengue case classification could be a model approach for other disease classifications.
26	Lin and others	2013	Review	SD	N/A	N/A	N/A	To compare the literature available on dengue case classification	N/A	D/SD has several advantages. First, by emphasizing dengue as a triphasic illness rather than as three distinct illnesses, physicians are reminded to observe their patients closely on a day-to-day basis for the appearance of WS until the critical phase has passed. Second, D/SD highlights that patients without WS may develop SD, which can be fulminant and unpredictable. D/SD provides practical guidance on how to manage patients more appropriately when laboratory data are unavailable as well as for monitoring severity, so that very severe cases that do not fulfill the original criteria for DHF are not missed. Using D/SD, physicians could upgrade the case evaluation according to clinical severity of disease manifestations.	D/SD provides practical guidance and shows better categorization in accordance with disease severity. Therefore, more prospective databases need to be established based on D/SD, which will enable not only the nationwide epidemiologic surveillance of severe cases in endemic countries but also, international comparison of data to improve patient management.
27	Srikiatkhachorn and others	2011	Review; expert opinion	Comparing DF/DHF/DSS and D/SD	N/A	N/A	Thailand	To evaluate DF/DHF/DSS and D/SD	None	The 2009 clinical classification represents a significant departure from the prior classification. In contrast to the previous classification, which defines DHF as a clinical entity with plasma leakage as the cardinal feature that differentiates it from DF, the 2009 classification lists several clinical manifestations as qualifiers for SD. The improvement in dengue-associated mortality over the past decades has been based on the understanding of the natural history of plasma leakage in DHF, which occurs around the time of defervescence and coincides with the nadir of the platelet count. Delayed detection and treatment of plasma leakage is the major cause of organ failure, listed as severe manifestations in the 2009 classification. By listing severe organ involvement as a criterion for severity separate from plasma leakage, the revised classification places emphasis on isolated organ failure as a common and significant cause of dengue severity. On the basis of the statistics from the outpatient department at Queen Sirikit National Institute for Child Health in Bangkok in 2008, the new case definition would have qualified an additional 39,000 cases as probable dengue, whereas only 1m600 suspected dengue cases were detected using a positive tourniquet test result and leukopenia as screening tools. The revised classification also poses significant problems for dengue research. Because the revised classification is designed primarily as a case-management tool, less emphasis is placed on the underlying pathophysiology.	Despite its limitations, the DHF case classification has proved to be useful for advancing important observations on dengue disease pathogenesis, such as the importance of secondary dengue virus infection to the plasma leakage phenomenon, and has been instrumental in the development of treatment regimens that have saved numerous lives.
28	Akbar and others	2012	Expert opinion/letter to the publisher regarding ref. 18	Comparing DF/DHF/DSS and D/SD	N/A	N/A	N/A	D/SD compared with DF/DHF/DSS	N/A	We believe the revised case classification with its simplified structure will facilitate effective triage and patient management and also allow collection of improved comparative surveillance data.	Efforts are also being directed to development of tighter definitions of severe phenotypes for basic science research.
29	Farrar and others	2013	Expert opinion/letter to the publisher regarding ref. 15	Comparing DF/DHF/DSS and D/SD	N/A	N/A	N/A	D/SD compared with DF/DHF/DSS	N/A	The main objectives of the classification scheme are to improve case management by timely identification of severe or potentially severe cases and ensure that scarce resources are directed to those patients most in need. D/SD presents significant improvements over the DF/DHF/DSS system in two key areas: (1) it reflects disease severity in real time, and (2) it allows identification of a higher proportion of clinically severe cases. Unfortunately, however, we must also recognize that we are no nearer to elucidating the mechanisms responsible for the microvascular derangements that are the hallmark of SD or understanding the immune correlates of protection than we were more than 40 years ago. Another long-established dogma that may have contributed to this lack of progress is the belief that DF and DHF are two separate disease entities with distinct clinical characteristics. Careful observational studies now suggest that the major clinical manifestations (altered vascular permeability, thrombocytopenia, coagulation derangements, hepatic dysfunction) show considerable overlap between the two syndromes and indicate that dengue virus infection disrupts a number of different physiological systems to varying degrees in individual patients, influenced by both host and viral factors, with the relative prominence of the resulting abnormalities determining the final clinical phenotype.	Classification schemes need to reflect the contemporary epidemiology of the disease, be able to assess severity in real time, and be globally harmonized. DF/DHF/DSS was too complicated to use in clinical or public health settings but was not sufficiently precise for detailed pathogenesis studies. D/SD brings clarity, clinical and epidemiological use, and the potential for development of more precise definitions of clinical phenotype for pathogenesis studies.
30	Horstick and others	2013	Expert opinion/letter to the publisher regarding ref. 20	WS comparing DF/DHF/DSS and D/SD	N/A	N/A	N/A	WS in D/SD compared with DF/DHF/DSS	None	WS have been in use in clinical practice for a long time, despite the knowledge that their sensitivities and specificities are far from perfect. WS have been used by clinicians as an additional tool, and their power has been described using a framework of risk increase in those patients with a WS present over those patients with no WS present. The WS currently proposed are broad clinical signs that can occur in many diseases. Their usefulness in dengue has mostly relied on expert opinion, with the exception of a few studies that have tried to provide empirical evidence for their usefulness. Hence, a prospective study design is of paramount importance to provide high-quality data.	Studies about WS need to be prospective and should include primary care to address the question of how WS can be used in the context of SD.
31	Wiwanikit and others	2013	Expert opinion/letter to the publisher regarding ref. 13	Comparing DF/DHF/DSS and D/SD	N/A	N/A	N/A	D/SD comparison with DF/DHF/DSS	Expert opinion	D/SD is exactly useful for grouping of the patient for additional management. However, whether the classification is useful for predicting of severity and outcome is still controversial.	D/SD can be applicable, which does not mean the classification is easy. There are many parameters to be collected, which might take time.
Other (excluded after full application of all inclusion and exclusion criteria as assessed from the text)											
32	Gupta and others	2010	Prospectively collected dengue cases	DF/DHF/DSS	Small	Prospective	India	In this prospective study of dengue infection during an epidemic in India in 2004, we applied the WHO classification of dengue to assess its usefulness for our patients	145 clinically suspected cases of dengue infection of all ages; dengue confirmation by IgM ELISA and HI test, and DF/DHF/DSS were applied to classify	Of 50 serologically positive cases of dengue enrolled in the study, only 3 met the WHO criteria for DHF, and 1 met the criteria for DSS; however, 21 (42%) cases had one or more bleeding manifestations.	By using WHO criteria of DHF on Indian patients, all severe cases of dengue cannot be correctly classified. A new definition of DHF that considers geographic and age-related variations in laboratory and clinical parameters is urgently required.
33	Srikiatkhachorn and others	2010	Prospectively collected database, retrospective data analysis	DF/DHF/DSS	Large	Retrospective analysis in an existing prospectively collected dataset	Thailand	Assessing if DHF criteria can identify SD cases, as determined by the requirement for fluid replacement and blood transfusion; sensitivity and specificity analysis of each component of DF/DHF/DSS	Sensitivity and specificity analysis for case definitions and several criteria	Our study showed that DHF is correlated strongly with the need for intervention. DHF constituted 68% of dengue cases that received significant intervention. However, 42% of DHF cases did not require intervention. In contrast, 15% of DF and 12% of OFI cases did require significant intervention. This finding shows the heterogeneity in severity in each disease category. In this study, 10 (76%) of 13 dengue cases with documented narrow pulse pressure were classified as DHF by the case definitions. Two of three patients with DF with hypotension had significant hemorrhage and required blood transfusion. Significant discordance between the grading of DHF cases by the expert and strict WHO criteria was also noted.	The current classification (DF/DHF/DSS) system seems to be suitable.
34	Wieten and others	2012	Prospectively collected dengue cases	Comparing DF/DHF/DSS and D/SD in non-endemic setting	Small	Prospective	The Netherlands with global travelers	The aim of this study was to assess the applicability and benefits of D/SD in clinical practice for returning travelers	Specificity and sensitivity and assessing the usefulness in predicting the clinical course of the disease	DF/DHF/DSS, compared with D/SD, had a marginally higher sensitivity for diagnosing dengue. D/SD had a slightly higher specificity and was less rigid. D+WS was admitted more often than those patients who had no WS (relative ratio = 8.09; 95% confidence interval = 1.80–35.48). Ethnicity, age, hypertension, diabetes mellitus, or allergies were not predictive of the clinical course	For returned travelers, D/SD did not differ in sensitivity and specificity from DF/DHF/DSS to a clinically relevant degree. The guidelines did not improve identification of severe disease.

The table shows included and excluded studies according to the study type. Large, > 500 cases; medium, 100–499 cases; small, < 100 cases. AST = aspartate transaminase; NS1 = non structural protein 1; CXR = chest X ray; N/A = not applicable; ITU = intensive therapy unit; IgM = immunoglobulin M; ELISA = enzyme-linked immunosorbent assay; HI = hemagglutination inhibition; OFI = other febrile illnesses.

**Table 2:** Reported sensitivities and specificities for dengue severity

Ref.	Authors	Place	Pro/Retro	Sample size	D/SD (%)		DF/DHF/DSS (%)		Comments
					Sensitivity	Specificity	Sensitivity	Specificity	
11	Basuki and others	Indonesia	Pro	145	88	99	74	100	Using a “clinical intervention tool”
13	Prasad and others	India	Pro	56	98	NR	24.8	NR	Comparing with “treatment levels”
16	Narvaez and others	Nicaragua	Retro	544	92.1	78.5	39.0	75.5	Comparing with “clinical intervention levels”
18	Siles and others	Peru	Retro	92	59	41	0	98	Comparing with “hospital level of care”
21	Van de Weg and others	Indonesia	Retro	173	70.5	79.5	89.9	25	Comparing with “intensive treatment intervention”

NR = not reported; Pro = prospective; Retro = retrospective.
